# An ultra-compact particle size analyser using a CMOS image sensor and machine learning

**DOI:** 10.1038/s41377-020-0255-6

**Published:** 2020-02-12

**Authors:** Rubaiya Hussain, Mehmet Alican Noyan, Getinet Woyessa, Rodrigo R. Retamal Marín, Pedro Antonio Martinez, Faiz M. Mahdi, Vittoria Finazzi, Thomas A. Hazlehurst, Timothy N. Hunter, Tomeu Coll, Michael Stintz, Frans Muller, Georgios Chalkias, Valerio Pruneri

**Affiliations:** 1 ICFO- Institut de Ciències Fotòniques, The Barcelona Institute of Science and Technology, 08860 Castelldefels (Barcelona), Spain; 2 Ipsumio B.V., High Tech Campus, 5656 AE Eindhoven, Netherlands; 3 Department of Photonics Engineering, Technical University of Denmark, DK-2800 Kgs Lyngby, Denmark; 4 Research Group Mechanical Process Engineering, Institute of Process Engineering and Environmental Technology, Technische Universität Dresden, Münchner Platz 3, D-01062 Dresden, Germany; 5 School of Chemical and Process Engineering, University of Leeds, Leeds, LS2 9JT UK; 6 IRIS Technology Solutions, SL, 08860 Castelldefels (Barcelona), Spain; 7 ICREA- Institució Catalana de Recerca i Estudis Avançats, 08010 Barcelona, Spain

**Keywords:** Imaging and sensing, Optics and photonics

## Abstract

Light scattering is a fundamental property that can be exploited to create essential devices such as particle analysers. The most common particle size analyser relies on measuring the angle-dependent diffracted light from a sample illuminated by a laser beam. Compared to other non-light-based counterparts, such a laser diffraction scheme offers precision, but it does so at the expense of size, complexity and cost. In this paper, we introduce the concept of a new particle size analyser in a collimated beam configuration using a consumer electronic camera and machine learning. The key novelty is a small form factor angular spatial filter that allows for the collection of light scattered by the particles up to predefined discrete angles. The filter is combined with a light-emitting diode and a complementary metal-oxide-semiconductor image sensor array to acquire angularly resolved scattering images. From these images, a machine learning model predicts the volume median diameter of the particles. To validate the proposed device, glass beads with diameters ranging from 13 to 125 µm were measured in suspension at several concentrations. We were able to correct for multiple scattering effects and predict the particle size with mean absolute percentage errors of 5.09% and 2.5% for the cases without and with concentration as an input parameter, respectively. When only spherical particles were analysed, the former error was significantly reduced (0.72%). Given that it is compact (on the order of ten cm) and built with low-cost consumer electronics, the newly designed particle size analyser has significant potential for use outside a standard laboratory, for example, in online and in-line industrial process monitoring.

## Analysis: Novel design uses machine learning for characterising materials

Scientists have developed low-cost particle size analyser (PSA) for conducting quality control and product testing in a range of industries, including the pharmaceutical, food and cosmetics. Currently, laser diffraction PSAs that use static light scattering to measure the size distribution of particles from hundreds of nanometres to several millimetres are the most widely used technology. However, these devices are generally large, expensive and complicated to operate, making them impractical for many industrial applications. Led by Valerio Pruneri from ICFO-The Institute of Photonic Sciences in Spain, a team of researchers has made a low-cost, miniaturised PSA capable of determining the volume median diameter of particles suspended in liquids. The novel design incorporates a collimated beam configuration using a commonly available image sensor to capture scattering images and machine learning to predict the particle size distribution.

## Introduction

Particle size analysis based on light scattering has widespread application in many fields, as it allows relatively easy optical characterisation of samples enabling improved quality control of products in many industries including pharmaceutical, food, cosmetic, polymer production, etc.^1–3^. Recent years have seen many advancements in light scattering technologies for particle characterisation. For submicron particle measurement, dynamic light scattering (DLS)^4^ has now become an industry standard technique. This method analyses the fluctuations of scattered light by particles in suspension when illuminated with a laser to determine the velocity of the Brownian motion, which can then be used to obtain the hydrodynamic size of particles using the Stokes-Einstein relationship. Although DLS is a useful approach to determine the size distribution of many nano- and biomaterials systems, it does suffer from several disadvantages. For example, DLS is a low-resolution method that is not suitable for measuring polydisperse samples, while the presence of large particles can affect the size accuracy^4^. Other scattering techniques have emerged, such as nanoparticle tracking analysis (NTA)^5^, which tracks individual particle movement through scattering using image recording. NTA also measures the hydrodynamic size of particles from the diffusion coefficient but is capable of overcoming some of the limitations posed by DLS^5,6^.

While the above-mentioned techniques are best suited for measuring particles typically in the submicron region, particle size analysers (PSAs) based on static light scattering or laser diffraction (LD)^7,8^ have become the most popular and widely used instruments for measuring particles from hundreds of nanometres to several millimetres. Similar scattering theory is also utilised in systems based on non-electromagnetic wave propagation, such as ultrasonic analysers^9,10^. In LD PSAs, a laser beam is used to irradiate a dilute suspension of particles. The light scattered by the particles in the forward direction is focused by a lens onto a large array of concentric photodetector rings. The smaller the particle is, the larger the scattering angle of the laser beam is. Thus, by measuring the angle-dependent scattered intensity, one can infer the particle size distribution using Fraunhofer or Mie scattering models^11,12^. In the latter case, prior knowledge of the refractive index of the particle being measured as well as the dispersant is required.

Commercial LD PSAs have gained popularity due to their broad dynamic range, rapid measurement, high reproducibility and the capability to perform online measurements. However, these devices are generally large in size (~700 × 300 × 450 mm), heavy (~30 kg) and expensive (in the 50–200 K€ range). On the one hand, the large size of common devices is due to the large distance needed between the sample and the detectors to provide the desired angular resolution. Furthermore, their high price is mainly due to the use of expensive laser sources and a large number of detectors, i.e., one sensor for each scattering angle to be monitored. Some commercial devices contain up to twenty sensors. This complexity of commercial LD PSAs, together with the fact that they often require maintenance and highly trained personnel, make them impractical in the majority of online industrial applications, which require the installation of probes in processing environments, often at multiple locations.

The application of LD PSAs is also normally restricted to dilute suspensions. This is because the optical models used to estimate the particle size distribution (PSD) are based on a single scattering approximation. In practice, most industrial processes require measuring concentrated suspensions, where multiple scattering becomes a prominent effect. Multiple scattering in dense media leads to an underestimation of the particle size since the light scattered by the particles encounters diffraction points multiple times before reaching the detector, which in turn increases the apparent scattering angle^13^. To overcome this issue, LD PSAs require appropriate sampling and dilution systems, which increase capital investments and operational costs. Another approach is to apply multiple scattering correction models together with the optical models to compute the PSD. A large number of algorithms for multiple scattering correction can be found in the literature^14–16^. However, these algorithms typically require implementing a complex correction, which increases the computation time and is often not suitable for online measurements^16^.

An alternative approach to compute the PSD without the use of optical models and complex correction factors is to apply machine learning (ML) techniques^17^. Machine learning is a valuable tool that relies on pattern recognition to learn and adapt to changes in processes and provide reliable results. It has been previously shown that, given the concentration and the angular distribution of scattered light, ML models can predict particle size even at high concentrations^18,19^. Such optimisations open up new opportunities for the use of LD PSAs in industrial processes without the need for time-consuming and cumbersome sample preparation. However, the low integration level for multiple sensor configuration and high cost of current commercial LD PSAs still remain significant barriers for their widespread implementation in online industrial monitoring.

Particles have also been measured using imaging techniques. More specifically, lens-free imaging systems that use complementary metal-oxide-semiconductor (CMOS) image sensors can perform direct imaging of particle holograms^20^ or particle diffraction patterns^21^. These systems allow the measurement of individual particles, differentiating them by geometrical shape, and do not require a significant refractive index difference between the particles and the containing medium^22^. In this work, we propose a novel low-cost and miniaturised PSA in a collimated beam configuration using a CMOS image sensor and an ML model based on a random forest algorithm^23^. In contrast to other lens-free imaging systems using CMOS sensors, we analyse the angular distribution of scattered light from an ensemble of particles, similar to the LD PSA. The proposed PSA device enables the measurement of samples with high concentrations. The key innovation in our proposed device is a small form factor (5 mm diameter, 17 mm long) angular spatial filter (ASF) made with an array of holes with different diameters that are extruded from a polymer rod. Light collected from different sized holes is representative of a different set of scattering angles. Upon illumination of the target sample with a light-emitting diode (LED), the ASF allows characterising the angular dependence of the scattered light by performing angle-resolved cumulative light power measurements. The patented ASF technology^24^ enables setting a specific design for each working size range. The rest of the analyser consists of off-the-shelf consumer electronic products, such as a CMOS image sensor array and LED light source. This design significantly reduces the cost and size compared to those of commercial LD PSAs, which require several detectors to obtain an adequately resolved angular scattering distribution.

To validate the new PSA, glass beads of different size distributions were measured, ranging from 13 µm to 150 µm at several concentrations in liquid dispersions. The random forest algorithm enables overcoming the current understanding of the theoretical limitations due to multiple scattering, enlarging the working size range and application possibilities, especially for measurements in liquid. By analysing the raw ASF images obtained from the CMOS image sensor array, we show how multiple scattering becomes prominent at high concentrations depending on the particle size being measured and how the random forest algorithm can correct this issue. Thus, the proposed PSA has great potential to become a cost-effective and compact solution for a broad range of industrial applications.

## Results

### Design and fabrication of ASF

**Fig. 1:**
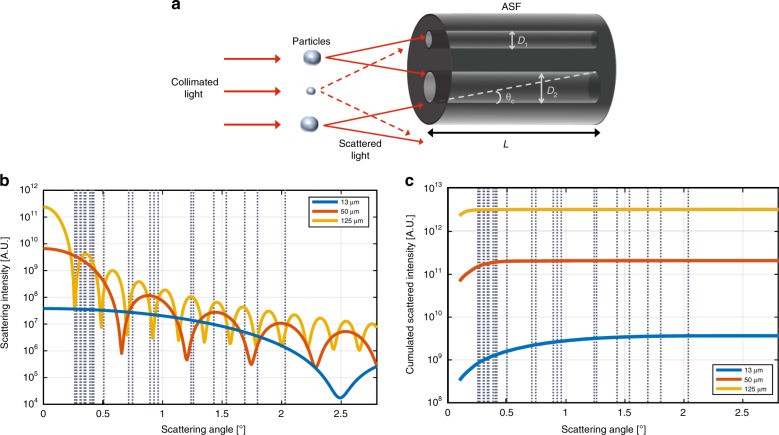
Concept of the new particle size analyser. **a** Schematic diagram of the ASF showing how the cut-off angle, *θ*_c_, is dependent on the diameter (*D*) and the length (*L*) of the holes. Light rays scattered from particles entering at angles larger than θ_c_ will be absorbed by the sidewalls. **b** The angular scattering profiles in water for three different glass beads of diameters 13, 50 and 125 μm with refractive index of 1.51 at a wavelength of 632.8 nm, simulated using the Mie algorithm^30^ in MATLAB. **c** Cumulative scattering intensity for the three particle sizes. Instead of sampling the scattering profile at each angle, the ASF apertures perform a cumulative scattering power measurement from zero to a predetermined *θ*_c_. The corresponding *θ*_c_ for each ASF hole for *L* = 17 mm, derived from Eq. 1 and converted to that in water using Eq. 2, is indicated by dashed vertical lines in **b** and **c**. We plot here results for single-particle scattering, but similar working principle description can be applied to the multiple-particle case

The ASF is the core of the proposed particle size analyser, which is capable of distinguishing different spatial frequencies scattered from the sample by means of a low-pass angular filter array. The ASF used in this work is an array of holes of different diameters that function as apertures (Fig. 1a). The angular acceptance— we call it the cut-off angle, *θ*_*c*_—for the scattered light of the apertures is determined by the hole’s diameter (*D*) and length (*L*):

1 $$\theta _c = \arctan \left( {\frac{D}{L}} \right)$$

The light scattered up to predefined *θ*_*c*_ values (shown with dashed vertical lines in Fig. 1b, c) is measured using a CMOS image sensor array that can simultaneously acquire power from multiple apertures (holes). This design allows the reconstruction of the cumulative angular scattering profile, as shown in Fig. 1c. In this description of the PSA and ASF working principle, we assume, for simplicity, that the inner walls of the ASF do not reflect, there is no crosstalk between the holes and the hole filtering has a square-like response up to the corresponding cut-off angle. In addition, Eq. 1 does not include effects on the calculation of *θ*_*c*_ due to light diffraction in the filter holes. In our work, as we will show later, there are instances where we observed residual reflection and diffraction effects through the ASF holes. However, the angular dependence of the ASF holes and the capability of the device to discriminate particle size and concentration are preserved. If necessary, light diffraction in the filter holes can be strongly reduced by increasing *D* and *L* proportionally, i.e., still maintaining the same *θ*_*c*_, as the typical diffraction angle is inversely proportional to D.

The larger the particle size is, the smaller the scattering angle is. Thus, a smaller *θ*_*c*_ is required, which means a larger *L/D* ratio. For example, to measure particles of hundreds of microns, we estimate that a minimum *L/D* ratio of 200 is required. For a typical length of several mm, this would mean a maximum *D* on the order of 50 µm. Making hole apertures with such dimensions and length is very challenging, even for the latest generation of 3D micro-printers using layer-by-layer fabrication. Other sophisticated techniques, such as mask-less photolithography, offer submicron resolution but cannot produce features with such high *L/D*. Additive manufacturing with micro-machining, e.g., laser sintering, selective laser melting and laser drilling, may achieve high *L/D* with micron resolution, but they impose significant constraints on the ASF, such as the combination of multiple pieces requiring tight alignment tolerances.

In this study, an interesting approach to overcome these fabrication hurdles and produce a highly optimised ASF including large arrays of holes with high *L/D* was to use a polymer extrusion technique. Such techniques have been widely used in fabricating micro-structured polymer optical fibres (mPOFs)^25^, for example. To construct the ASF, a micro-structured cane was fabricated using a drill-and-draw technique from a commercially available poly(methyl methacrylate) (PMMA) rod from Nordisk Plast. A cane preform was prepared by machining the rod to 60 mm in diameter and 100 mm in length, which was followed by drilling the desired hole patterns. The preform was then annealed for a week at 80 °C and drawn to canes of 5 mm in diameter and 50 mm in length. A complete description of the experimental methodologies involved in the drill-and-draw technique can be found in^26^. This method of fabricating the ASF allows high flexibility in design since both *D* and *L* for the holes can be easily adjusted to collect scattering angles required for specific applications.

The fabricated ASF used in this work consists of 23 holes with diameters ranging from 112 to 800 µm. The length is selected to be 17 mm so that the PSA incorporating such ASF can measure scattering angles from 0.38 to 2.7°. However, for measuring particles in suspension, these angles need to be corrected because the rays from the particles undergo refraction at the flow cell wall, i.e., water-glass and glass-air interfaces. The relation between the detected (*θ*_*c*_) and the actual (*θ*) scattering angles is given by:

2 $$\begin{array}{*{20}{c}} {\sin \theta = \frac{{\sin \theta _c}}{{n_w}}} \end{array}$$

where *n*_*w*_ is the refractive index of the water, giving *θ* from 0.29 to 2.02°. Using Mie theory, we can approximate this angular range of the current ASF to be suitable for measuring particles from approximately 10 to 125 µm. A smaller and larger hole diameter-to-length ratio is required to measure particles above and below this range, respectively. Note that for very small particles (i.e., below 10 µm), the signal-to-noise ratio becomes a limiting factor due to the weak scattering signal intensity. A more sensitive image sensor array, such as a commercially available single-photon camera, can be used to improve the measurement at low scattering intensity. The implementation of such a camera will be a topic of further study.

To account for the multiple scattering effect at high concentrations that causes widening of the scattering lobe, we polish one side of the ASF along the entire length. This process leaves an empty space inside the holder, which acts as a large aperture. Such an aperture allows the entire angular spectrum of the forward scattered light to be collected from the sample.

The mPOF polymer used for fabricating the ASF is only partly absorbing in the working wavelength range in the visible spectrum. The inner walls of the ASF are thus covered with a black acrylic paint to increase their absorption and reduce reflection and crosstalk between adjacent holes.

### Design of the PSA using the ASF

**Fig. 2:**
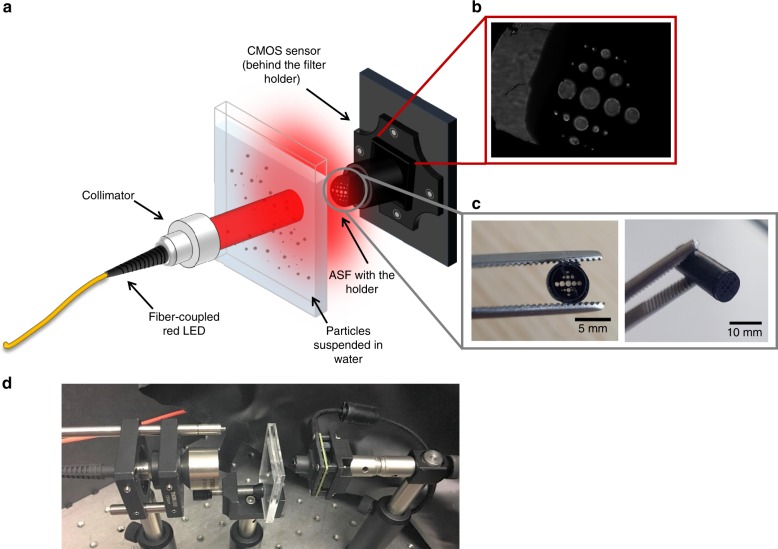
Design of the proposed PSA using the ASF. **a** Schematic diagram of the PSA with a novel ASF that allows angle-resolved forward scattering measurements, in combination with a CMOS image sensor array and a collimated LED source, **b** An example raw image of sample with a volume median diameter of 44 µm at a concentration of 15 mg ml^−1^ obtained from the CMOS image sensor array. **c** Photograph of the fabricated ASF and **d** laboratory prototype showing the compactness of the proposed PSA

Figure 2a depicts the schematic diagram of the proposed PSA design based on the ASF. A fibre-coupled and collimated red LED—with a wavelength of 632.8 nm—is used as the light source. A 10 mm beam illuminates the sample containing particles dispersed in water. The scattered and unscattered light from the sample is collected by the ASF and the holder attached to the CMOS image sensor array. Additional details on the CMOS can be found in the Materials and Methods section.

All the data analysis in this work is conducted using MATLAB and Python. A typical raw image from the CMOS image sensor is shown in Fig. 2b and the fabricated ASF in Fig. 2c. The corresponding lab prototype is shown in Fig. 2d.

### Measurements of particle suspensions

The experiments using the proposed PSA were carried out with samples listed in Table 1 for concentrations ranging from 1 to 40 mg ml^−1^. For the smallest particle size range, i.e., 13–20 µm, the highest concentration that could be measured was 10 mg ml^−1^, where above this concentration the light intensity reaching the CMOS image sensor array becomes too low and would require longer integration times to achieve reliable results. At the beginning of each sample measurement, 200 ml of water was circulated through the flow cell, and a set of five images was obtained with a time gap of between 20 and 60 s. For each concentration, a sample suspension was added from the stock solution (100 mg ml^−1^ concentration) to the water, and images were captured. The flow cell was cleaned with deionized water prior to measuring each new sample. A schematic diagram of the experimental setup is shown in Supplementary Fig. S1, and the raw images obtained from the CMOS sensor of the samples measured at a certain concentration are shown in Supplementary Fig. S2.

**Table 1:** Sample characteristics and concentrations measured.

Sample	Density (gcm^−3^)	Refractive index @ *λ* = 632.8 nm	Size range (µm)	Commercial LD PSA (HELOS/KR-H2487)			Concentrations measured (mg ml^−1^)
				D10 (µm)	D50 (µm)	D90 (µm)	
Guyson	2.5	1.51	80	55	74	92	1,5,10,15,20,25,30,40,50
			40	24	39	56	1,5,10,15,16,18,20,25,30
Cp5000	2.56	1.51	13–20	6	11.9	21	1,2,3,4,5,6,7,8,9,10
Sovitec	2.46	1.51	0–50	18	34.8	51	1,5,10,15,18,20,22,25,30,40
			40–50	33	43.6	51	
			40–70	46	62.3	80	
			70–110	68	87.5	108	
			90–150	97	125.5	157	

**Fig. 3:**
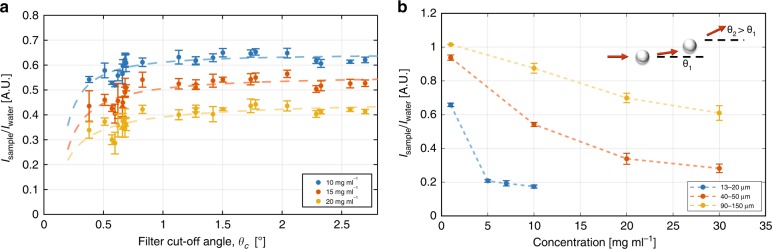
Measurements performed with glass beads at different concentrations. **a** The average intensities normalised to water of the filter holes for glass beads with a 40–50 µm diameter distribution are plotted as a function of filter cut-off angles (*θ*_*c*_)—calculated from the holes’ diameters and length of ASF using Eq. 1—for three different concentrations. The error bars represent the 95% confidence interval. The dashed lines guiding the eyes represent a least square fit. **b** The average intensity normalised to water of the 112 µm diameter hole against concentration for three different glass bead diameter distributions, 13–20, 40–50 and 90–150 µm. The dependence on concentration, increasing for smaller glass beads, is a signature of multiple scattering

The light distribution between the ASF holes depends on the concentration. In Fig. 3a, we show this dependence for glass beads with a 40–50 µm size distribution. The intensity values plotted are the average of the five images calculated using the ¨regionprops¨ function in MATLAB. For the same concentration, smaller particles present a larger effect on the measured intensity and its dependence on the scattering angle (see Figure S3). This phenomenon can be explained in terms of the multiple scattering effect, where particles undergo several scattering events before reaching the CMOS image sensor array^14–16^. The result is the widening of the scattering angle and hence a decrease in the forward scattering intensity. This finding is also confirmed by Fig. 3b, where the average intensity of a small hole for three different particle size distributions is plotted against particle concentration.

### Particle size prediction using a machine learning algorithm

While a high concentration leads to multiple scattering effects, an excessively low concentration leads to a poor signal-to-noise ratio. Therefore, a certain working concentration range must be defined for different particle size distributions. To avoid this concentration dependence and facilitate a wide working concentration range, we developed a machine learning algorithm using a random forest model, as explained within the Materials and Methods, to predict D50 from a given image and concentration value.

**Fig. 4:**
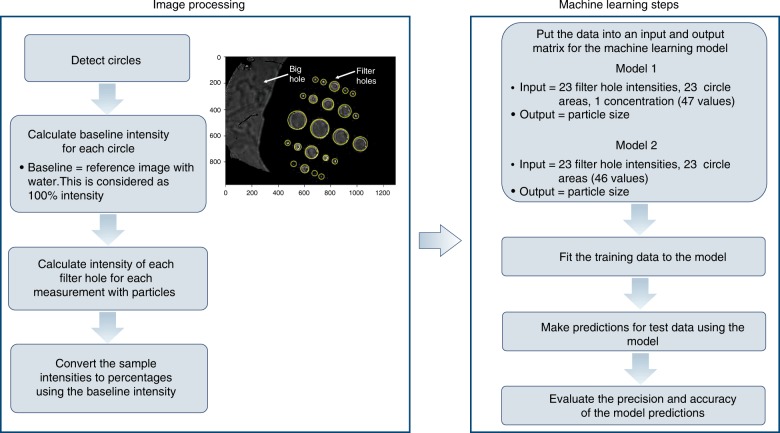
Flowchart of the particle size detection algorithm using machine learning

**Fig. 5:**
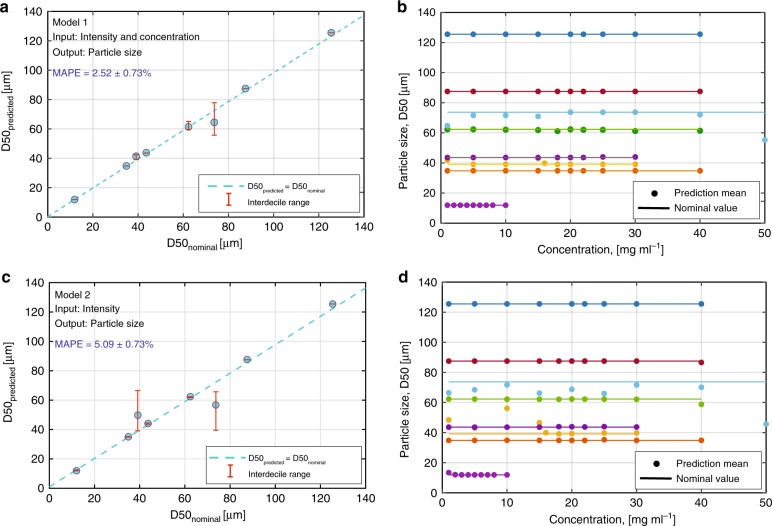
Performance of the machine learning algorithm in the prediction of particle size (glass bead diameter) when trained and tested with images obtained from the CMOS image sensor array. Two models are used for training and testing purposes. Model 1 used the intensity and diameter of the 23 holes together with the concentration information for training, whereas Model 2 used only the intensity and diameter (46 features) for inference. **a** The mean predicted D50 values using Model 1 for one of the test sets are compared to the nominal D50 values measured using a commercial LD PSA (HELOS/KR-H2487). The dashed line represents predicted diameter = nominal diameter. The interdecile range is also shown for each predicted D50. **b** The D50 prediction values from Model 1 are plotted against concentration. Despite the multiple scattering effects that produce a strong dependence on concentration, the predicted diameters are close to the nominal diameters (straight lines). **c** The mean predicted D50 against nominal diameter using Model 2 and **d** D50 prediction against concentration using Model 2. When only spherical particles were analysed, the error for Model 2 was significantly reduced from 5.09 to 0.72% (see Supplementary Fig. S6)

The image processing and the machine learning steps are summarised as a flowchart in Fig. 4. The mean and the standard deviation as a function of data points for one set of measurements were first monitored. After 100 repeats, no significant improvement in the predicted error was observed. Hence, the model was trained and tested 100 times. The mean of 100 mean absolute percentage error (MAPE) on the test sets was found to be 2.52%, with a standard deviation of 0.73%. The performance of the model on only one of these test sets is depicted in Fig. 5a, b. The random forest model can therefore correct the dependence on particle concentration that leads to the multiple scattering effect (Fig. 5b) and predict the particle size with high accuracy (Fig. 5a).

So far, we tested Model 1 to predict D50 using concentration as one of the inputs. Since in practice, the particle size should be provided as an independent parameter from concentration, we tested Model 2, which relies only on filter sizes and intensities to predict D50. Upon testing the model, the MAPE was found to be 5.09 ± 1.56%. The predicted D50 vs nominal D50 and D50 vs concentration plots are given in Fig. 5c, d, respectively. As expected, Model 1 has a higher precision than Model 2 when concentration information is given as input, but the prediction error without concentration is still acceptable.

We also performed separate training of the model using intensity values from the large hole only for all the particle sizes measured (Supplementary Fig. S4). Note that the big hole analysis is performed on the same images as the ASF holes. The large hole intensity includes the entire angular spectrum for the scattered light and scales as the absorption due to the particle solution. The model was tested 100 times with different test sets. The mean predictions were found to deviate significantly from the nominal values with varying concentrations, and the mean error was increased to 23.03 ± 5.61%. This result confirms that absorption analysis is not enough and that scattering and the ASF play crucial roles in predicting particle size with high accuracy using the random forest model. In addition, the big hole analysis indicates that there are no hidden correlations between the different images, other than those related to the particles (e.g., size, concentration) on which scattering depends. If there were hidden correlations, then the MAPE for the large hole would not have given such large errors compared to those obtained with the ASF analysis.

In particle size analysis, D50 is certainly one of the most important parameters. However, some applications require knowledge of the distribution, as given by the D10 and D90 parameters, which correspond to fine and coarse particles, respectively, in the sample. The same machine learning algorithm can also be trained to predict additional percentile values for the volume median diameter, e.g., D5, D10, D15 to D95, without the need to modify the experimental set-up. We performed a trial training of the random forest model with the D10, D50 and D90 values measured using a commercial LD PSA; on testing the model, the MAPE was found to be 4.27 ± 1.64%, 3.02 ± 1.07% and 2.4 ± 0.8%, respectively. Even though the results are quite promising with only one set of D10 and D90 data for each size, they can be further improved by measuring samples with the same D50 but varying distribution spread. Future development will include experiments with different refractive indices and different size range particles.

In addition to the above-mentioned batch measurements, we performed a test flow-through measurement (described in supplementary information) to demonstrate the capability of our ML model for such measurements. We collected data with two samples, 13-20 µm and 40-70 µm, for different concentrations and calibrated our previous model with these data. We then tested the model on a new set of data for the same samples collected on a separate day. The MAPE for Model 1 was found to be 1.77 ± 0.25% (Supplementary Fig. S5). Though only two samples were measured, this preliminary result suggests that our system can be used to predict the change in particle size for different samples. With further optimisation, the flow measurement procedure and performance can be improved, and the accuracy can be increased.

We also note that larger deviations from the nominal value are observed for the Guyson beads (D50: 39 and 74 µm). Microscope images (see Supplementary Fig. S6a and b) of these beads reveal the presence of some non-spherical particles, the shape of which has an influence on their scattering pattern. Supplementary Fig. S6c and d shows the performance of Model 2 for all glass beads except the Guyson beads and for only the Guyson beads, respectively, confirming that the MAPE is strongly increased by the Guyson beads. By removing the particles with a non-spherical shape from the sample analysis, the MAPE becomes much smaller (0.72%). Therefore, our device performs according to ISO13320^27^, which requires that for polydisperse spherical particles, the measured D50 should be within 2.5% of the quoted maximum or minimum values for the reference materials. In future work, by collecting more data, including on non-spherical particles, one can expect that the precision of the device will increase further.

## Discussion

In this work, we proposed a novel design of a compact, portable and cost-effective particle size analyser (PSA) in a collimated beam configuration using a CMOS image sensor and machine learning. Unlike commercially available counterparts, such as laser diffraction-based systems that use several detectors to measure the scattering signature of particles, the proposed PSA uses an innovative design including a novel, very small angular spatial filter. The ASF combined with an LED and a CMOS image sensor array allows the acquisition of angle-dependent scattering images that are used by an ML model to predict the median diameter of particles.

The proposed PSA was validated by measuring glass beads of various size distributions at different concentrations. The results obtained from the ML model showed that, given the particle concentration, the median particle size could be measured, with a low mean absolute percentage error of 2.5%, even in the presence of significant multiple scattering. When the concentration is not an input parameter, this error increases to 5%. However, by removing samples with non-spherical particles, we achieve a MAPE of 0.72% for Model 2, i.e., without predefining concentration as an input parameter. These performances compare well with those of commercially available laser diffraction-based counterparts, with reported device accuracies for monomodal latex standards of approximately 0.6%.

While future improvements in the optical hardware and a larger quantity of data for the ML algorithm, including non-spherical particles collected with well-designed sample feeding systems for dry and wet measurements, will lead to higher precision, we intend to utilise the inherent flexibility of the simple design and low hardware cost of our proposed PSA for incorporation in online or at-line applications. For online operations, such instrumentation is mostly used for quality assurance (QA) and control purposes, which are often focused on measuring system changes rather than necessarily exact values. We have shown such an example of a real-time change response with the proof-of-concept measurements in a flow cell system using our proposed PSA. As such, the performance is a trade-off between the hardware cost and the required level of accuracy, where the exact error limits will likely not be as low as those for commercial *ex situ* PSAs. The additional benefit of online analysis is the lower degree of sample intrusion, and thus, for many particle processes, measurements are often actually more representative of the system, even if the absolute instrument error is higher.

Therefore, we believe our proposed PSA is an attractive solution for online monitoring of particles in different industrial processes without the need to perform dilution operations. We also note that our proposed PSA device is sensitive to the refractive index difference between the particles and the surrounding medium. In principle, the system may thus also be used in relevant biological applications, for example, in the detection of microorganisms in water, such as *Escherichia coli* and *Legionella*, and red cells in blood.

## Materials and methods

### Glass bead characterisation

To test the functionality of the newly designed PSA, we measured various size distributions of glass beads at different concentrations, which are summarised in Table 1.

The sample suspensions in water at each concentration are measured using a commercial LD PSA (Model HELOS/KR-H2487, Sympatec GmbH, Clausthal-Zellerfeld, Germany) for angular ranges below 35° (i.e., forward scattering). In this work, an angular range of 0.1° to 9° is used since this range is sensitive to particle sizes from 0.5 μm to 175 μm^28^. The volume median diameter D50 together with the volume-weighted 10th and 90th percentiles, D10 and D90, respectively, of the particle distributions for each sample measured with HELOS/KR-H2487 are also listed in Table 1.

Electron micrographs of the particles dispersed in water are also taken using a scanning electron microscope (SEM), model DSM 982 Gemini (Zeiss/Germany). The SEM is a low-voltage electron microscope (30 kV) with a maximum magnification of 200,000, i.e., a resolution of approximately 10 nm. The instrument detects both types of electrons, namely, backscattered primary and backscattered secondary electrons, and therefore can provide high sizing accuracy and three-dimensional impression.

The particle size distributions obtained from the HELOS/KR-H2487 system together with the SEM images of the glass beads used in the experiments are shown in Supplementary Fig. S7.

### Particle suspension preparation

For each size range to be measured, a known mass of powder samples is taken and dispersed in a known volume of deionized water to make a suspension. An overhead stirrer at 300 rpm is used to prevent agglomeration or deposition of the particles in the beaker containing the suspension. The suspension is then circulated into a flow cell (component of HELOS-KR-SUCELL—Sympatec GmbH, Clausthal-Zellerfeld, Germany; measurement volume ~ 6 ml) with a path length of 4 mm using a peristaltic pump. The pressure of the pump is controlled to prevent air bubble formation while maintaining a homogeneous flow of suspension in the measurement cell.

### Image acquisition and processing

The CMOS image sensor array used to capture the images is a Micron MT9P0311, and the images are displayed using DevWare software. The active area of the image sensor array is 5.7 × 4.28 mm = 24.4 mm^2^, which is also the field-of-view of the proposed PSA when the ASF is in close proximity to the flow cell; it consists of 2592 × 1944 pixels, each of which has a size of 2.2 × 2.2 µm. The array has four colour channels, of which only red is used for data processing in our experiments. The frame rate to obtain a full-resolution image is 14 frames per second (fps).

Prior to each measurement, a dark image in the absence of LED illumination is captured and subsequently subtracted from the sample images. For each particle size range, first, a reference image with only water is measured, and then, images of suspensions at different concentrations are measured.

### Machine learning algorithm

The data from the sensor include an image per test condition (concentration and standard particle size). The following procedure was developed to correlate the images to the median volume particle size D50 used in each test. First, the location of the 23 filter holes is established using an image processing library (scikit-image, blob detection) in Python. The pixel intensities and diameters are calculated for each hole. The pixel intensities are then converted to relative intensities, as percentages, using the reference images to which pixel intensities of 100% are assigned. A set of five images is obtained for each combination of concentration and particle size, and the average of these images corresponds to a single data point: (a) the known concentration and D50 and (b) the relative intensities and diameters for 23 holes. Second, the dataset comprises 459 images that are randomly partitioned into two sets: (a) the training set (344 images) and (b) the test set (115 images).

Third, the random forest algorithm is used to find the correlation between the input variables (the concentration, the 23 relative intensities and 23 hole diameters) and the output variable (D50). Among the different ML algorithms available^29^, we chose the random forest because it is suitable for structured data, as in our case. We have also made a preliminary comparison between gradient boosting and random forest and confirmed that the latter provides slightly better predictions for the number of data points used in the analysis. The random forest consists of multiple decision trees. Each tree is a tree-like model of decisions. Each decision (splitting of the data) uses one feature and its threshold value. Learning (i.e., training) includes choosing the features, threshold values and when to stop the tree. The model is developed using a scikit learn machine learning library, with the hyper-parameters given in Supplementary Table S1.

Fourth, the generation of the training/test sets is a random process; therefore, model performance can change from split to split. To handle this fluctuation, steps 2 and 3 are repeated 100 times. MAPE of model predictions on the test set is used to assess the performance of the model. It is defined as:

3 $$\begin{array}{*{20}{c}} {{\mathrm{MAPE}} = \frac{{100\% }}{n}\mathop {\sum }\limits_{i = 0}^n \left| {\frac{{{\mathrm{Actual}}\,{\mathrm{value}}_i - {\mathrm{Predicted}}\,{\mathrm{value}}_i}}{{{\mathrm{Actual}}\,{\mathrm{value}}_i}}} \right|} \end{array}$$

where *n* is the number of images in the test set.

The mean MAPE of 100 models and their standard deviations are reported as the final figure of merit. To visualise the predictions of one of the models on the test set, predictions are plotted per particle size.

Until now, we developed a model that predicts D50 using concentration as one of the inputs. We refer to this as Model 1. For a truly functional sensor, however, predicting D50 only from intensity, without any input concentration, is essential. Therefore, we developed another random forest model, Model 2, which uses only filter sizes and intensities (i.e., 23 hole diameters and 23 intensity values) to predict D50.

## Supplementary information

Supplementary information for An ultra-compact particle size analyser using a CMOS image sensor and machine learning
