# Supplementary material for: An ultra-compact particle size analyser using a CMOS image sensor and machine learning
Source: Light Sci Appl. 2020 Feb 12;9:21. doi: 10.1038/s41377-020-0255-6 (PMC7016131; doi:10.1038/s41377-020-0255-6)
Supplement: Supplementary file 1 — Supplementary information for An ultra-compact particle size analyser using a CMOS image sensor and machine learning [file 41377_2020_255_MOESM1_ESM.docx]

# Supplementary information for

# An ultra-compact particle size analyser using a CMOS image sensor and machine learning

**Authors:** Rubaiya Hussain^1^, Mehmet Alican Noyan^1,2^, Getinet Woyessa^3^, Rodrigo R. Retamal Marín^4^, Pedro Antonio Martinez^1^, Faiz M. Mahdi^5^ ,Vittoria Finazzi^1^, Thomas A. Hazlehurst ^5^, Timothy N. Hunter^5^, Tomeu Coll^6^, Michael Stintz^4^, Frans Muller^5^, Georgios Chalkias^6^, Valerio Pruneri^*1,7^

**Affiliations:**

^1^ICFO- Institut de Ciències Fotòniques, The Barcelona Institute of Science and Technology, 08860 Castelldefels (Barcelona), Spain.

^2^Ipsumio B.V., High Tech Campus, 5656 AE Eindhoven, Netherlands

^3^Department of Photonics Engineering, Technical University of Denmark, DK-2800 Kgs. Lyngby, Denmark.

^4^Research Group Mechanical Process Engineering, Institute of Process Engineering and Environmental Technology, Technische Universität Dresden, Münchner Platz 3, D-01062 Dresden, Germany.

^5^School of Chemical and Process Engineering, University of Leeds, LS2 9JT, UK.

^6^IRIS Technology Solutions, SL, 08860 Castelldefels (Barcelona), Spain.

^7^ICREA- Institució Catalana de Recerca i Estudis Avançats, 08010, Barcelona, Spain.

**Corresponding author^*^: Prof. Valerio Pruneri**

**Email:** [**valerio.pruneri@icfo.eu**](mailto:valerio.pruneri@icfo.eu)

**
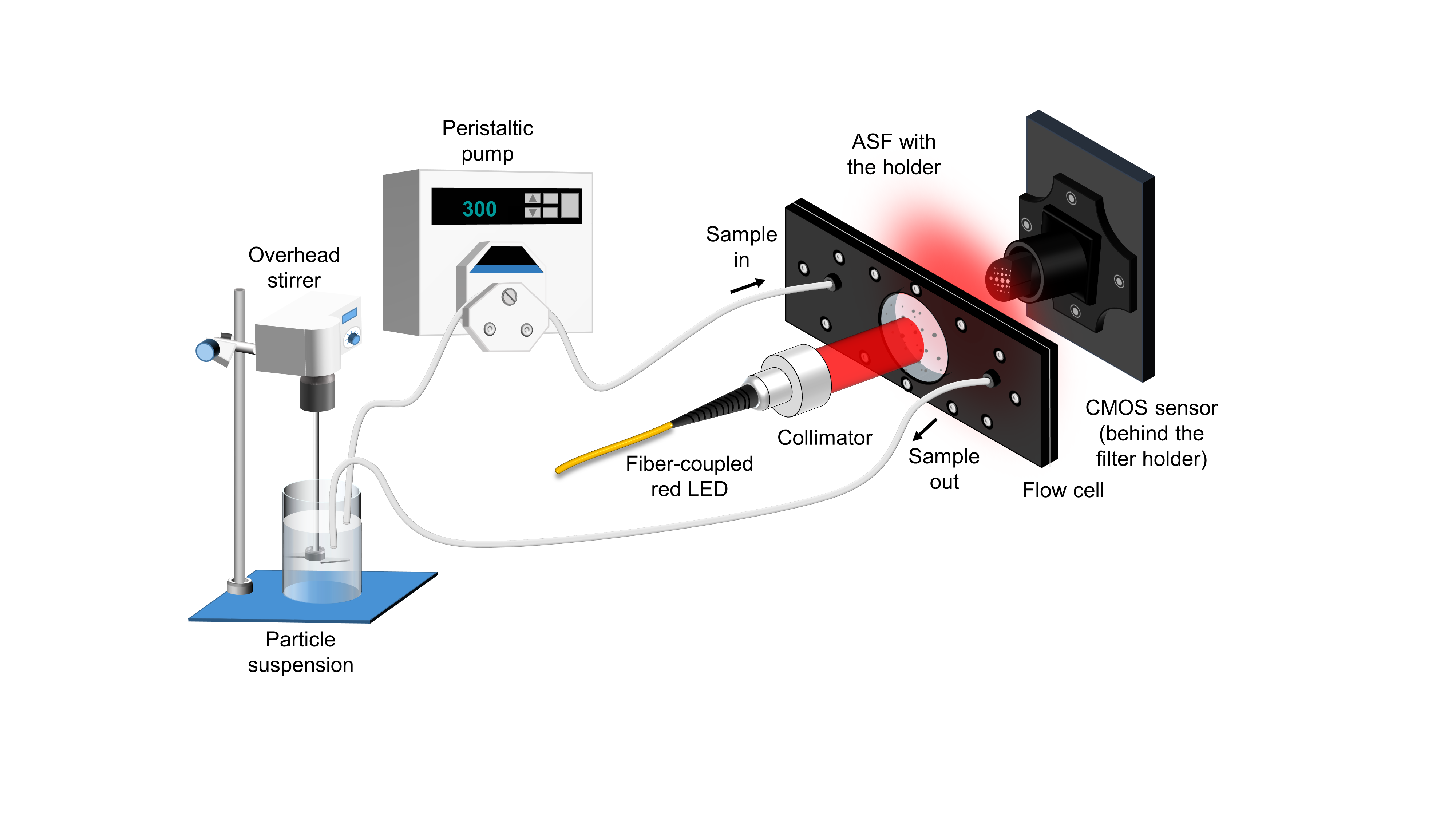
**

**Figure S1. Experimental setup.** Schematic diagram of the experimental setup showing the flow cell for circulating the particle suspension, the overhead stirrer and the peristaltic pump.

**
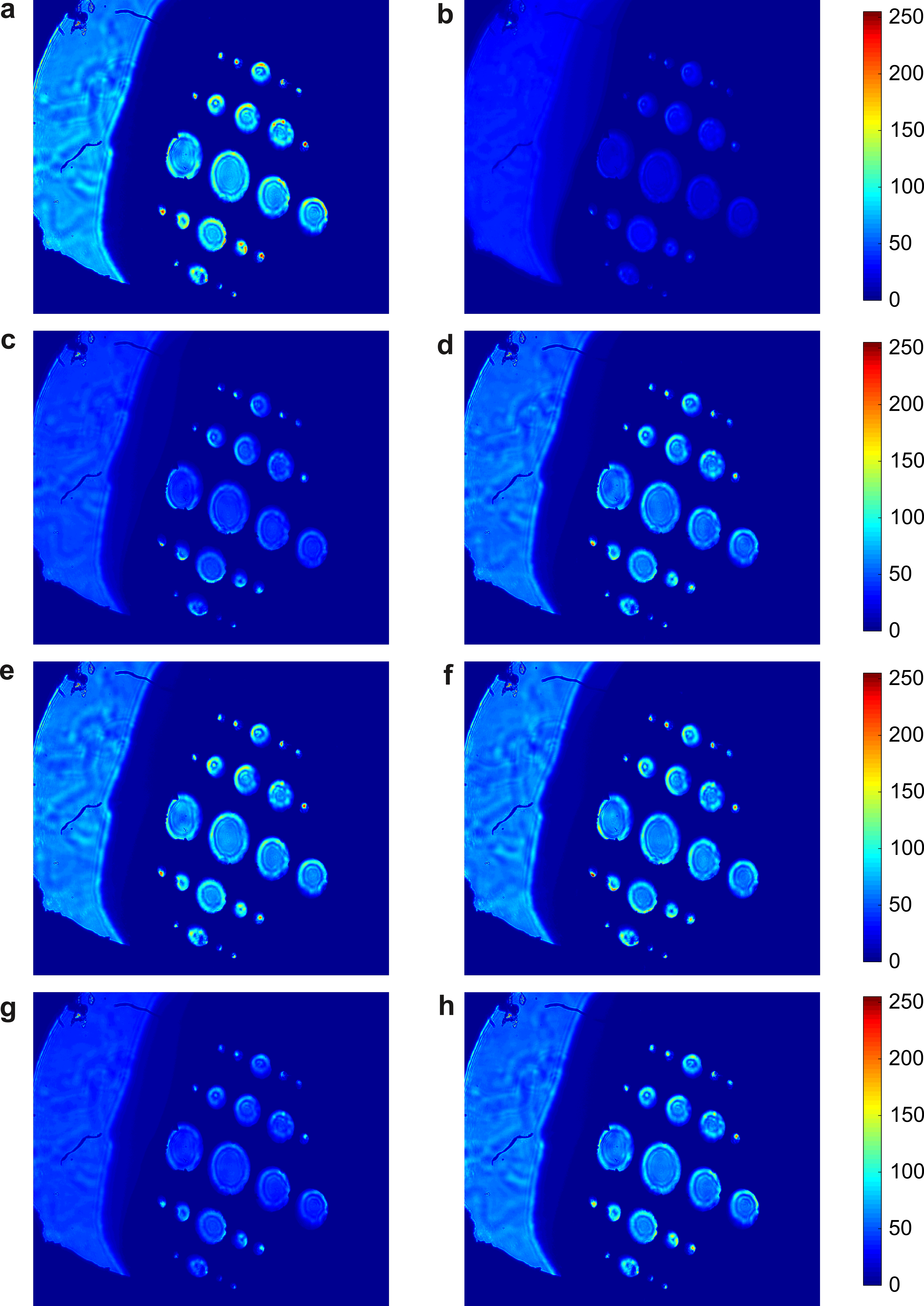
**

**Figure S2. Raw images of measured particles at 10 mgml^-1^ unless otherwise stated**. a) water, b) Cp5000 13-20 µm at 5 mgml^-1^ concentration, c) Sovitec 0-50 µm, d) Sovitec 40-70 µm, e) Sovitec 70-110 µm, f) Sovitec 90-150 µm, g) Guyson 40 µm and h) Guyson 80 µm. In some holes the images show local intensities deviating from the corresponding average values. We believe that these are likely to be associated to residual diffraction and reflection from the inner part of the holes’ walls and geometrical and material imperfections of the ASF (e.g. non-perfect hole geometry, slight misalignment between holes and missing black paint). Despite these imperfections and associated effects, the ASF angular dependence of the transmission of each hole is maintained, the ASF is still very efficient in discriminating different particle sizes and concentrations (see figure 3 in the main text and figure S3 below) and the proposed PSA device still provides high accuracy after using machine learning algorithm.

**
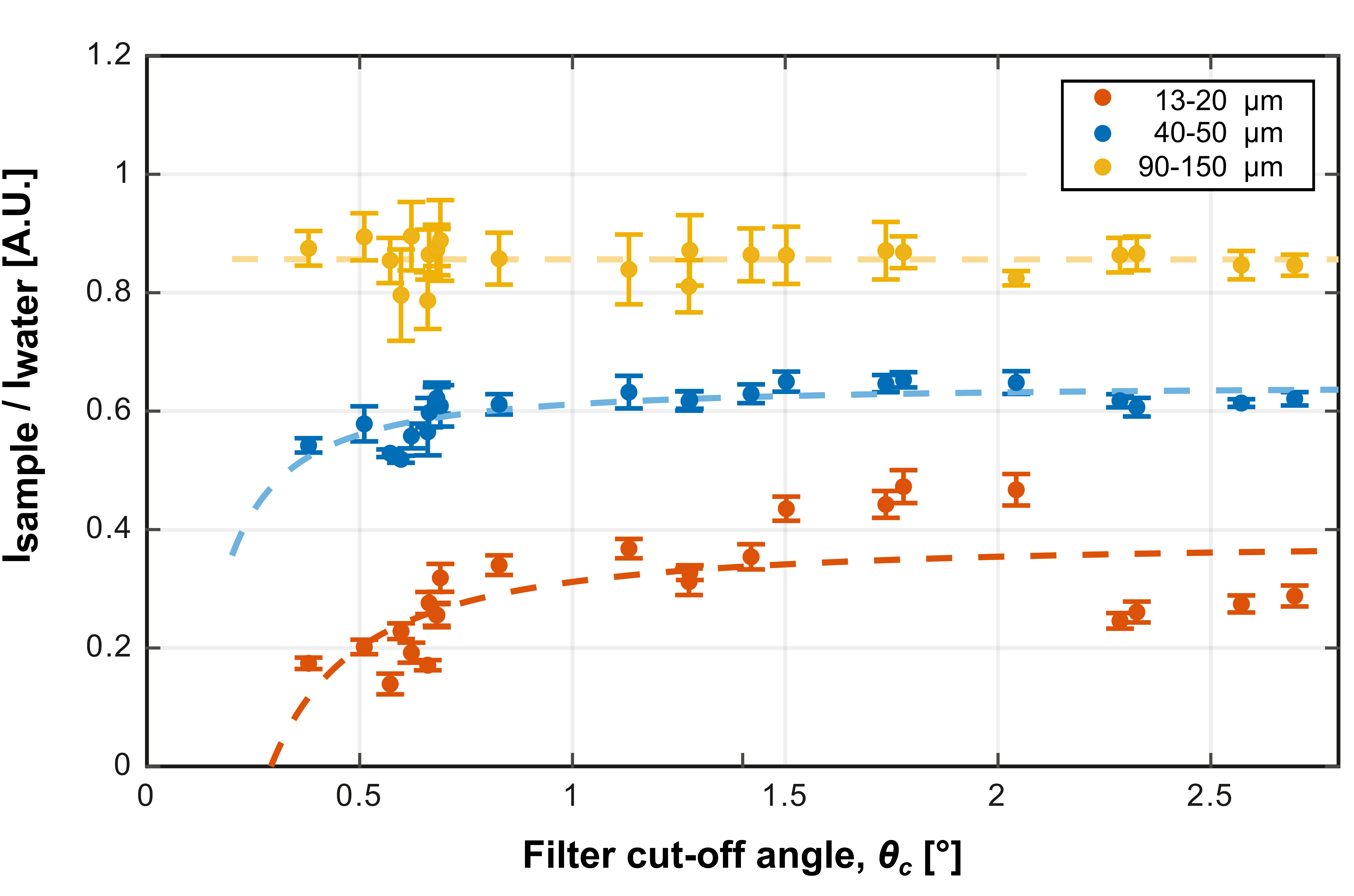
**

**Figure S3. Measurements with glass beads.** The average intensities of the small filter holes are plotted as a function of filter cut-off angles (*θ_c_*), for the three ranges of glass bead diameters and same concentration (10 mgml^-1^). Error bars represent 95% confidence interval.

**
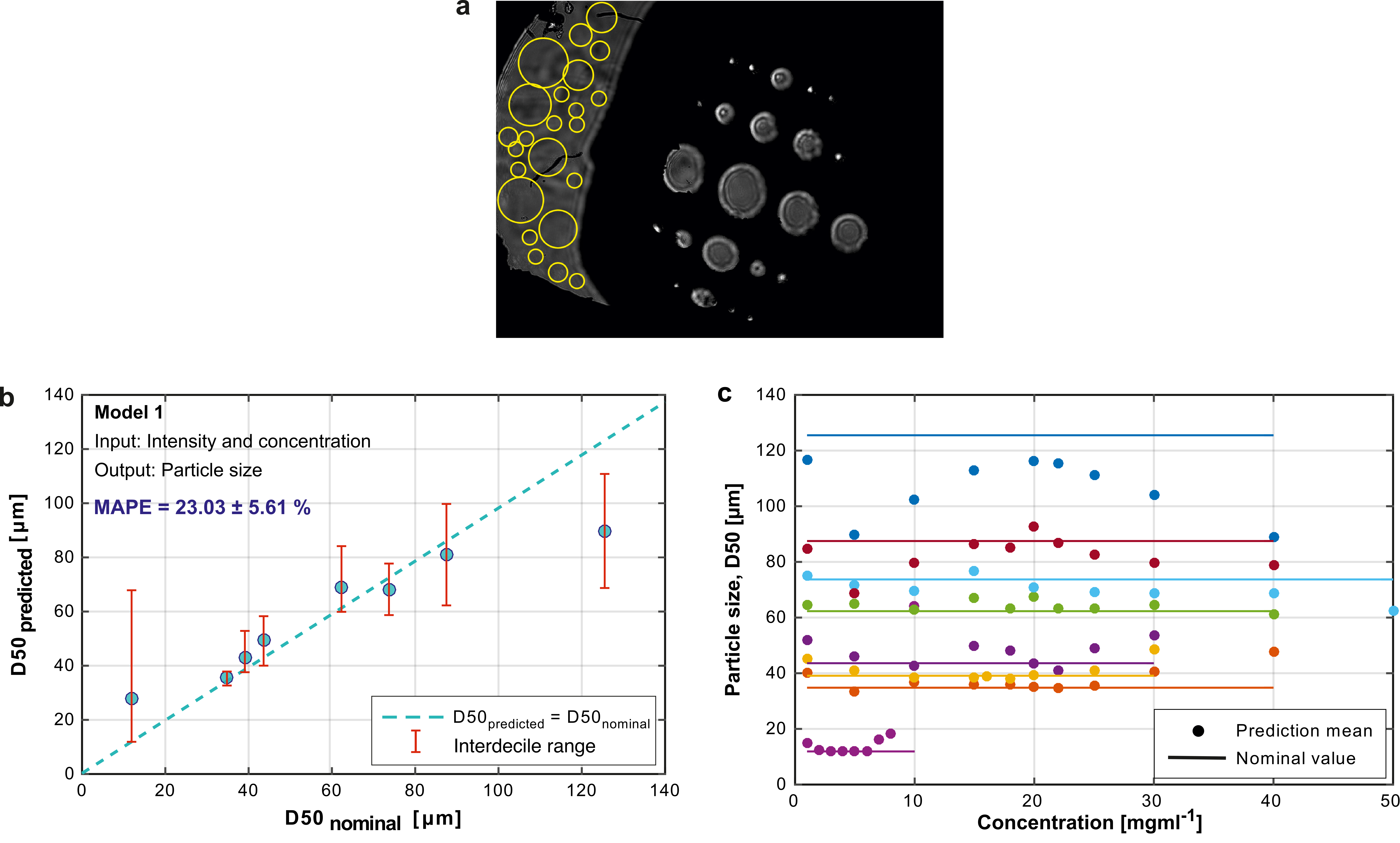
**

**Figure S4. Testing the model with big hole intensity and concentration as input.** The model requires 47 input values. So to train and test the model we used intensity values from the big hole within regions defined by the 23 filter hole areas. The yellow circles in a) represents the 23 filter holes placed in a random manner. The mean predictions against nominal values for one of the test sets are shown in b) while against concentration in c). The deviations of predictions with respect to nominal values are much larger than those obtained with the ASF filter (Figure 5a in main article), clearly indicating that scattering and ASF filter are required for the machine learning model to predict particle size with high accuracy.

**Flow-through Measurements**

In order to demonstrate the capability of our machine learning model for flow-through measurements, we performed preliminary tests with two samples, 13-20 µm (Sample 1) and 40-70 µm (Sample 2). In the first trial series, Sample 1 was measured at a concentration of 2 mgml^-1^, followed by Sample 2 at 5 mgml^-1^ without cleaning the flow cell. The same experiment was repeated on the same day, at a different time. For the second trial series, Sample 1 was measured at three different concentrations, one after the other, followed by Sample 2, again at three different concentrations one after the other. The same experiment was then repeated, where this time, Sample 2 was flowed first and then Sample 1. The previous machine learning Model 1 was then calibrated with three of these new data sets and tested on a 4th data set. The prediction result is shown in Figure S5.

**
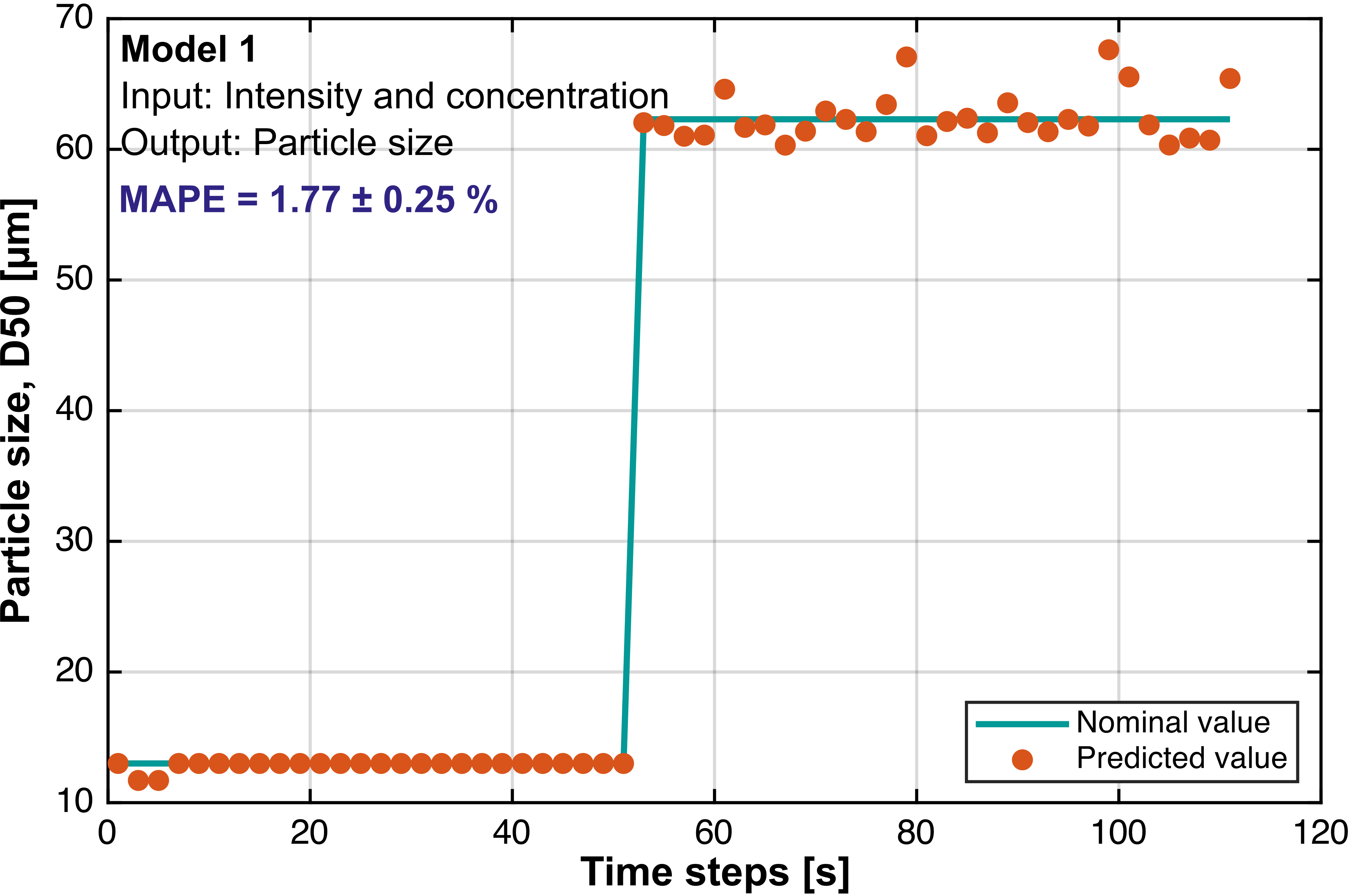
**

**Figure S5. Flow-through measurements.** Two samples, 13-20 µm (D50 13 µm) and 40-70 µm (D50 62.3 µm), are measured continuously one after the other without cleaning the flow cell. The MAPE for Model 1 is found to be 1.77 % with a standard deviation of 0.25%. This preliminary result suggests that the hardware and model are capable of predicting the median volume diameter change for flow-through measurements. Note that with the current set-up the transition from one particle size to another is too fast to capture a sufficiently large number of images to properly train the ML model. This is the reason why the graph does not contain a transitory region where both particle sizes coexist.


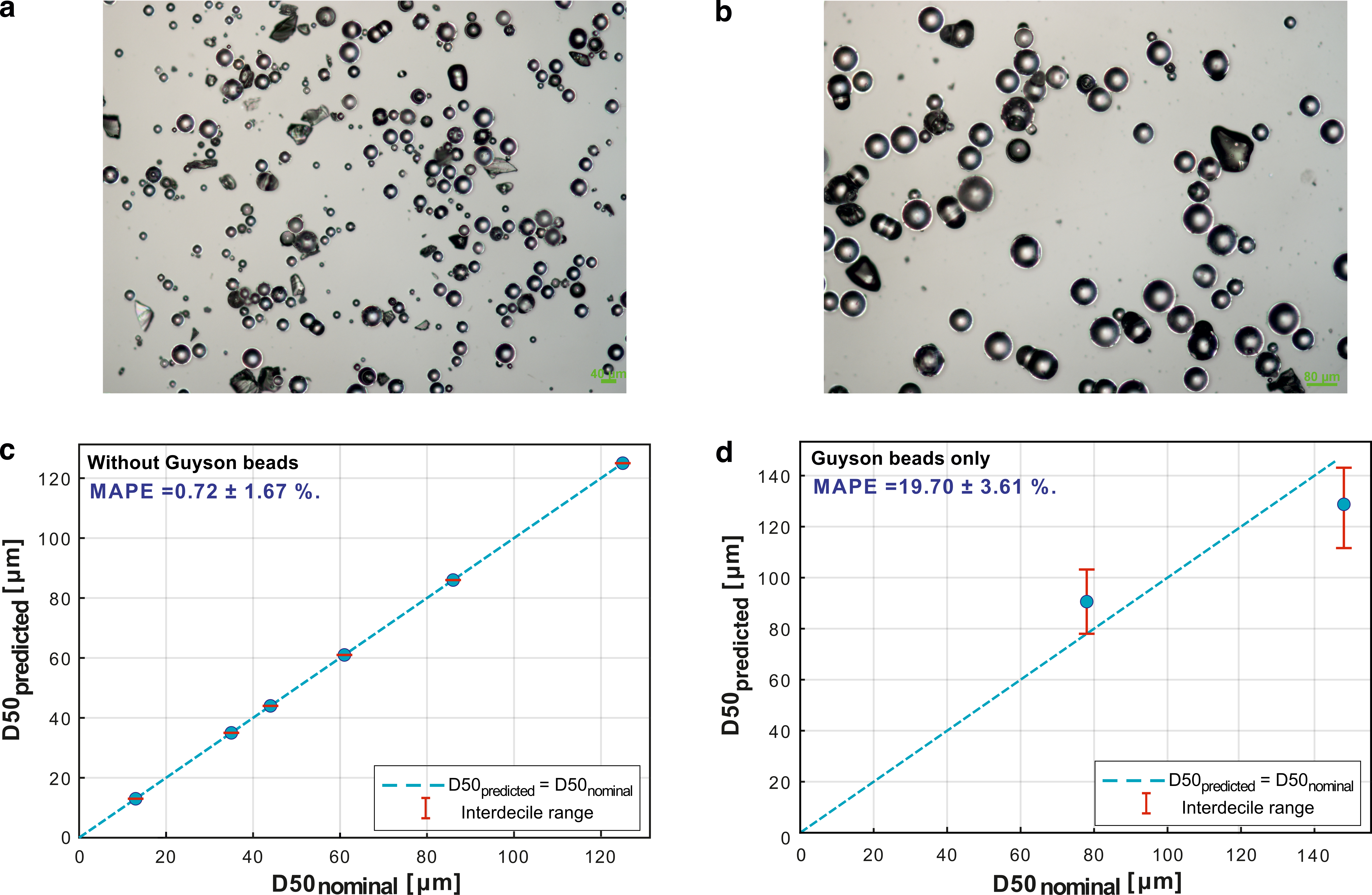


**Figure S6.** **Guyson beads**. Microscope image of Guyson beads with D50 a) 39 µm and b) 74 µm show the presence of some non-spherical particles. Performance of Model 2 c) without Guyson beads and d) with Guyson beads only. It can be seen from c) that when the model is trained and tested without Guyson beads the model performance improves significantly whereas in d) with only Guyson beads, the MAPE is seen to increase.

**
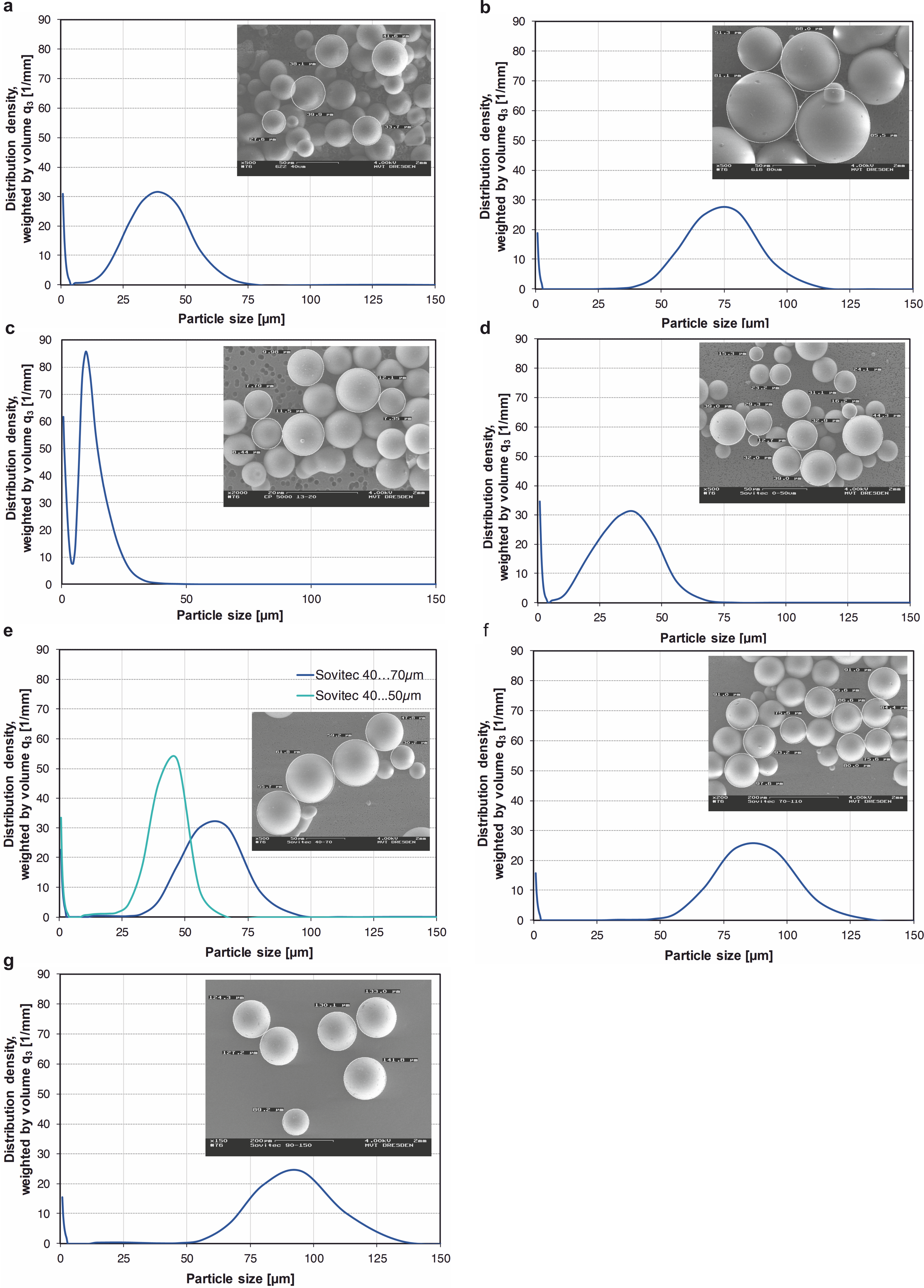
**

**Figure S7. The PSD and SEM micrograph of measured particles of various size distributions.** a) Guyson 40 µm b) Guyson 80 µm c) Cp5000 13-20 µm d) Sovitec 0-50 µm, e) Sovitec 40-70 µm and 40-50 µm, f) Sovitec 70-110 µm and e) Sovitec 90-150 µm.

**Table S1.** Hyper-parameters used for the Random Forest Regressor (sci-kit learn).

| Bootstrap = True | Max leaf nodes = None | Min samples split = 2 | Oob score = False |
| --- | --- | --- | --- |
| Criterion = mse | Min impurity decrease = 0.0 | Min weight fraction leaf = 0.0 | Random state = 0 |
| Max depth = 20 | Min impurity split = None | N estimators = 100 | Verbose = 0 |
| Max features = “auto” | Min samples leaf = 1 | N jobs = None | Warm start = False |
